# Isolated tear of the cord-like middle glenohumeral ligament in Buford complex: A case report

**DOI:** 10.1097/MD.0000000000008604

**Published:** 2017-11-10

**Authors:** Byung-Ill Lee, Yong Beom Kim, Sung Hun Won, Shu Chiang Hwang, Sung-Woo Choi, Jae-Hwi Nho, Dong-il Chun

**Affiliations:** a Center of Arthroscopy; b Department of Orthopaedic Surgery, Soonchunhyang University Hospital Seoul, Seoul, Korea.

**Keywords:** Buford complex, isolated tear, middle glenohumeral ligament, shoulder

## Abstract

**Rationale:**

We describe a rare case of the isolated tear in the cord-like middle glenohumeral ligament (MGHL) in Buford complex. To the best of our knowledge, this is the first report in the English literature about the isolated tear of cord-like MGHL in Buford complex. The present report describes in detail our experience with the diagnosis of isolated tear of the cord-like MGHL in Buford complex and management.

**Patient concerns:**

A 50-year-old female patient visited our hospital with pain and stiffness in the right shoulder that lasted for 9 months.

**Diagnoses:**

The clinical impression was frozen shoulder by primary impingement syndrome of right shoulder and the magnetic resonance (MR) images showed tear of cord-like MGHL.

**Interventions:**

We repaired the torn MGHL with the arthroscopic technique.

**Outcomes:**

Positive outcome by arthroscopic repair demonstrates that this disease entity is one of spectrum of pathologic condition in shoulder joint.

**Lessons:**

Isolated tear of cord-like MGHL should be restored to the original anatomy for positive outcome.

## 1 Introduction

The middle glenohumeral ligament (MGHL) has the greatest variation in its shape and size among all the ligaments of the shoulder joint. The Buford complex, a kind of normal anatomic variant, has been defined as the combination of the absence of the anterior superior labrum and the presence of a cord-like MGHL, which was not found frequently.

Recent advances in diagnostic capabilities such as magnetic resonance (MR) arthrography and arthroscopy allowed us to have more information about glenohumeral ligament complex (GHLC) and to differentiate normal variants from pathologic disorders of shoulder joint. The common normal variations of the GHLC are the sublabral foramen, the cord-like MGHL, and the Buford complex.

We describe a rare case of the isolated tear in the cord-like MGHL in Buford complex and the clinical result after arthroscopic repair of the torn cord-like MGHL.

## 2 Case description

This case report was approved by the Institutional Review Board of Soonchunhyang University Hospital.

**Figure 1:**
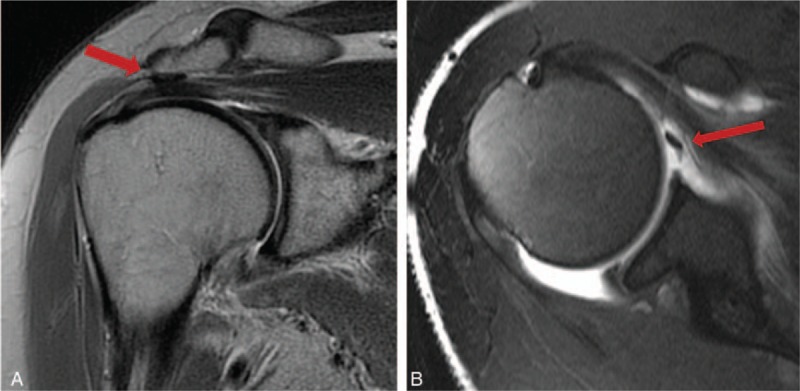
A, Preoperative T2-wighted MR image of soft tissue super in subacromial space (arrow). B, Preoperative MR arthrogram image of ruptured cord like MGHL. MGHL = glenohumeral ligament, MR = magnetic resonance.

**Figure 2:**
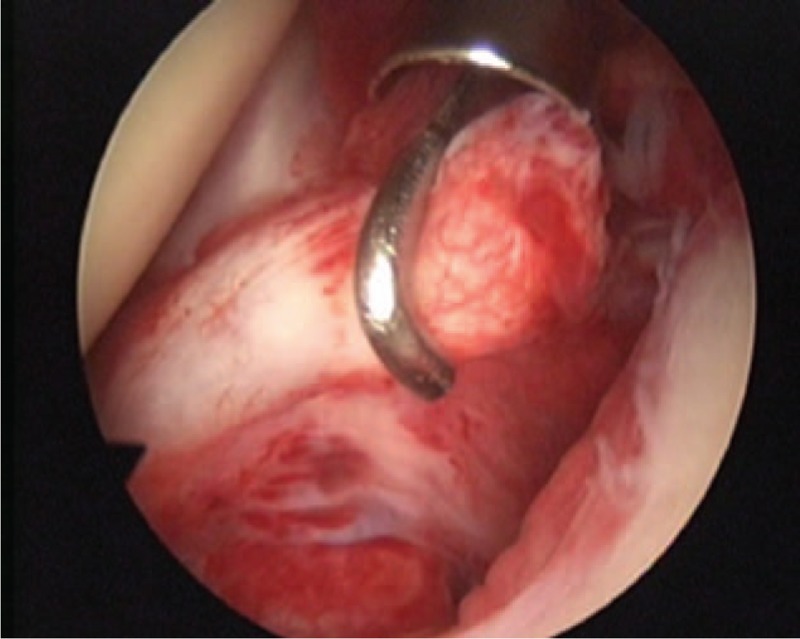
Arthroscopic view of a right shoulder from a posterior portal. The cord-like MGHL is discontinuous with the superior labrum. Also, the absent anterior superior labrum is shown, illustrating the Buford complex. MGHL = glenohumeral ligament.

**Figure 3:**
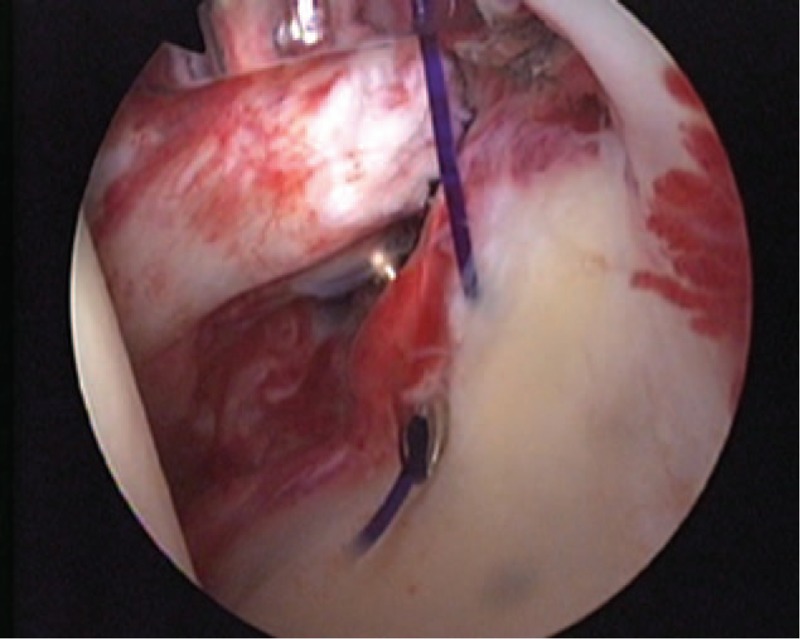
Arthroscopic view demonstrated the repair procedure of cord-like MGHL. A suture hook loaded with a monofilament suture was used to penetrate the stump of cord-like MGHL and superior labrum. MGHL = glenohumeral ligament.

**Figure 4:**
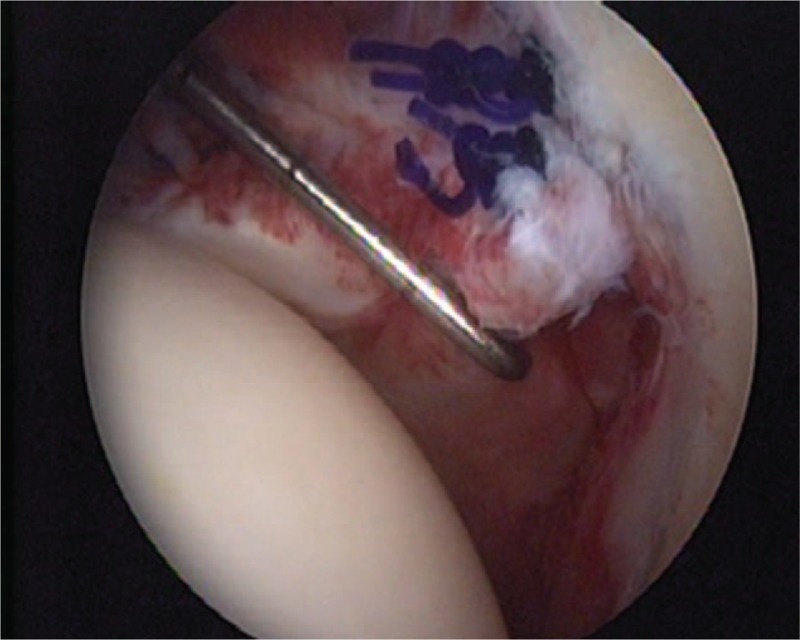
Arthroscopic view demonstrated the repaired MGHL with 2 No. 1 PDS sutures. MGHL = glenohumeral ligament, PDS = polydioxanone suture.

A 50-year-old female patient visited our outpatient clinic with pain and stiffness in her right shoulder that lasted for 9 months. One year ago she had been injected with steroid injection in her subacromial space of right shoulder, and the pain was subsided. But 9 months ago, the pain was recurred in the same side. The pain was refractory to medications and physical therapy. The pain was more severe at night and during particularly abduction and external rotation of shoulder. On initial physical examinations, the active range of motion (ROM) of abduction (<45°), external rotation (<10°), internal rotation (<15°), and forward flexion (<45°) was decreased due to the pain. The impingement signs (Neer and Hawkins–Kennedy impingement sign) were positive. The MR images showed mild subacromial bursitis and formation of small bony spur at the anterior undersurface of the acromion (Fig. 1A). Under the clinical impression of frozen shoulder by primary impingement syndrome of right shoulder, we decided to perform the surgical treatment. On arthroscopic findings, there was slightly injected synovium of capsule, a cord-like MGHL stump suggesting tear and absence of anterosuperior glenoid labral tissue (Fig. 2), and there were injected bursa, small spur in subacromial space, soft tissue fraying of subacromial surface, but no impinged sign of coracoacromial ligament. On retrograde review of MR, we could confirm the findings suggesting the tear of cord-like MGHL (Fig. 1B). The injected synovium was debrided and the stump of torn cord-like MGHL was checked and reduced to the original site. After reduction of original architecture, we repaired the torn MGHL with suture hook and 2 No. 1 Polydioxanone Suture sutures (Figs. 3 and 4). After the operation, an arm sling brace was applied to protect against abduction and external rotation of shoulder. Pendulum passive motion exercise was begun the day after the operation within range of painless motion. After 2 weeks of pendulum exercise, the shoulder pain was decreased and the exercise of passive and gentle active ROM and the wall climbing exercise were performed. Three months after the operation, working activities were allowed and the patient was satisfied with decreased pain and improved function. This patient underwent passive assisted ROM exercise for 6 weeks after the operation and daily living ROM exercise for 3 months, which resulted in pain-free outcome with full ROM at postoperative 6 months. The physical examination including the measurement of the ROM revealed (abduction, external rotation, internal rotation, forward flexion, respectively), 50°, 30°, 30°, 60° at postoperative 6 months, 100°, 30°, 40°, 90° at postoperative 12 months, and 130°, 50°, 60°, 140° at postoperative 24 months. On the final follow-up of postoperative 2 years, the University of California, Los Angeles score, Rowe score, and ASES score were 28/35, 90/100, and 87/100 respectively.

## 3 Discussion

Variations of normal anatomic structures in the glenohumeral joint are common and must be distinguished from pathologic conditions. The anterior GHLC was readily observed and consists of the labrum and glenohumeral ligaments (superior, middle, anterior-inferior). The common variations of anterior MGHL include a sublabral foramen, cord-like MGHL, and the Buford complex.^[1,2]^ The incidence is reported to be 8% to 12% for the sublabral foramen, 1.5% to 5% for the Buford complex, and 19% to 23% for the cord-like MGHL. Buford complex was initially introduced as a rare normal variant with little clinical significance.^[2]^ The criteria for the diagnosis of Buford complex followed the original description and included the visualization of a distinct cord-like MGHL originating directly from the superior labrum and crossing the subscapularis tendon to insert on the humerus and nonvisualization of the anterior superior labrum with a normal appearance of the anterior inferior labrum.^[2]^

But Huber and Putz^[3]^ described a periarticular fiber system, consisting of the labrum, glenohumeral ligaments, and inserting tendons. They suggest that the system of parallel collagen fibers surrounding the circumference of the glenoid acts as a tension brace to provide hoop stresses at the periphery.^[3,4]^ Bents and Skeete^[1]^ presented the Buford complex with an absent anterior labral tissue and suggested that it allows abnormal stresses to the superior labrum and biceps and predisposes the patient to a possible Superior labrum anterior and posterior lesion. They emphasized the possibility of superior labral lesion in the patient with a Buford complex. Although there was a report about isolated MGHL injury of 33 patients, the report dealt with the avulsion of the labrum from glenoid in all the cases except 1 case.^[2]^They suggested that the mechanism of injury was either repetitive overhead activity or a forced hyperextension in neutral rotations of arm abducted between 45° and 90°.^[2,5]^ This position could stress the MGHL resulting in the initiation of tearing in the level.

## 4 Conclusion

Isolated tear of cord-like MGHL in Buford complex is not found frequently. Our case showed an isolated tear of cord-like MGHL that was injured at the origin of the cord-like MGHL and absence of anterior superior labral tissue consistent with a Buford complex. The surgical procedures (arthroscopic repair) used for the patient were just designed to restore the original anatomy of the cord-like MGHL. Our experience of this positive outcome by arthroscopic repair demonstrates that this disease entity is one of spectrum of pathologic condition in shoulder joint. Since there was no accumulated data of this type of injury, we could not assure the validity of this procedure. Further studies are needed to confirm the pathologic prognosis of this disorder and also to confirm the effectiveness of surgical repair.
